# An infant of KID syndrome along with Dandy-Walker malformation (DWM)

**DOI:** 10.1016/j.jdcr.2025.10.031

**Published:** 2025-11-06

**Authors:** Avik Mondal

**Affiliations:** Department of Dermatology, All India Institute of Medical Sciences (AIIMS), Kalyani, West Bengal, India

**Keywords:** alopecia, Dandy-Walker malformation, ichthyosis, KID syndrome, sensory neural hearing loss

## Introduction

Keratitis-Ichthyosis-Deafness (KID) syndrome is a rare keratinization disorder due to missense mutation of the GJB2 gene (connexin 26) or, rarely, GJB6 (connexin 30).^1^, ^2^, ^3^, ^4^, ^5^ Clinical features include ichthyosiform scaling, palmoplantar hyperkeratosis, stippled keratoderma, alopecia along with bilateral hearing loss, and keratitis with corneal neovascularization.^1^, ^2^, ^3^, ^4^, ^5^ All changes may not be seen at a time; it takes time to appear at or before puberty.^1^ Here, I present a rare case of KID syndrome in an infant that is associated with Dandy-Walker malformation (DWM).

## Case presentation

A 5-month-old boy, born out of non-consanguineous parents, presented with alopecia of the scalp and eyebrows along with generalized dry scaling. Parents gave a history of recurrent hospital admission due to recurrent upper respiratory tract infection and pustular eruption over the scalp and trunk. The child had an uneventful birth history and no similar family history. No history of photophobia or sluggish response to sound was found.

**Fig 1:**
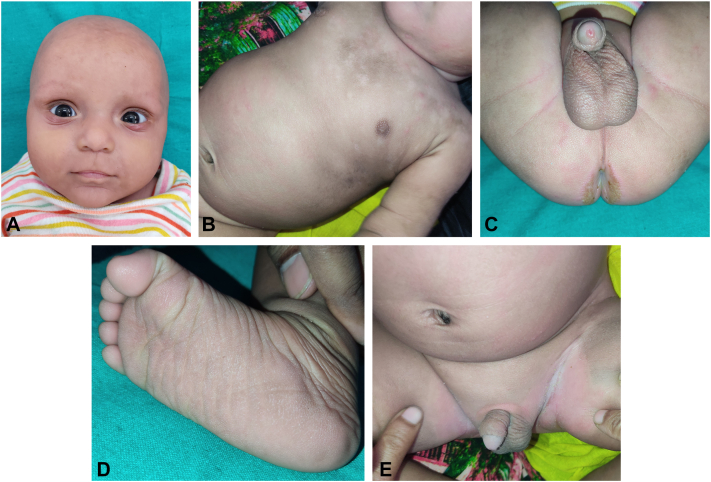
**A,** Alopecia over scalp, eyebrows, lower eyelid sparing upper eyelid. **B,** Involvement of trunk in the form of ichthyosis with erythema and brownish adherent scaling. **C,** Hyperkeratotic *brownish plaque* surrounding anus. **D,** Stippled keratoderma of right sole. **E,** Symmetrical involvement of bilateral groins in the form of erythematous macerated plaque.

On examination, the child had alopecia over the scalp and eyebrow sparing the upper eyelid. Generalized xerosis with some areas having brownish adherent scaling. The perianal region showed hyperkeratotic plaque with erythema. Bilateral groins revealed macerated, ill-defined erythematous plaques. Palmoplantar keratoderma with a stippled pattern was seen (Fig 1, *A*-*E*). Nail and mucosae didn’t reveal any abnormality.

Initially, I kept atrichia due to vitamin-D receptor gene mutation, IFAP (ichthyosis follicularis, alopecia, and photophobia) syndrome, and primary immunodeficiency as differentials. I did an extensive workup (complete blood count, liver, renal function, quantitative immunoglobulin analysis, T-lymphocyte enumeration, HIV serology, total hemolytic complement, vitamin D, and gene sequencing done from a blood sample). All were normal except gene analysis (whole exome sequencing) that revealed heterozygote pathogenic mutation in the single coding exon 2 of the *GJB2* gene (p.Asp50Asn), which encodes connexin 26.

After getting the genetic report, KID syndrome was the final diagnosis, and to find out if there was any hearing loss, brainstem-evoked response audiometry was done, which revealed bilateral severe to profound sensory neural hearing loss. An ophthalmological examination was performed, revealing no abnormalities of the cornea. The otolaryngologist provided the child with hearing aids before evaluating the option of cochlear implants. From a dermatological perspective, the patient was prescribed acitretin (0.50 mg/kg/d).

**Fig 2:**
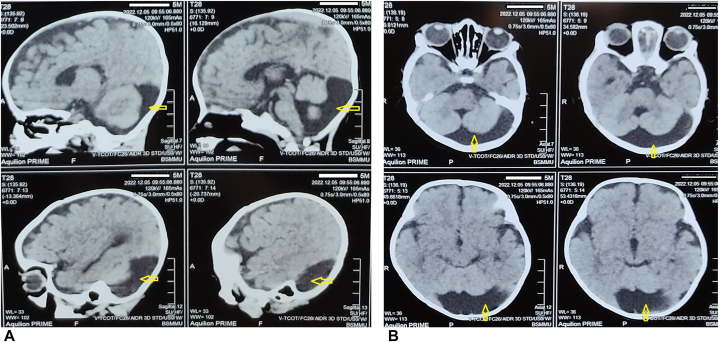
(**A,** Sagittal section) and (**B,** Coronal section): Hypoplasia of the cerebellar vermis, dilatation of the cisterna magna and the fourth ventricle on NCCT brain (*yellow arow*).

Prior to discharge, a routine pediatric consultation was conducted, which indicated the child's developmental delay. Incidentally, I came across a case series of DWM observed in KID syndrome. Following a pediatric neurology consultation, a non-contrast computed tomography (NCCT) of the brain was performed, revealing hypoplasia of the cerebellar vermis, as well as dilatation of the cisterna magna and the fourth ventricle, findings consistent with DWM (Fig. 2, *A* and *B*).

During acitretin treatment, he exhibited substantial resolution of skin lesions and flattening of the skin on his palms and soles, observed at the 3-month follow-up. He is also under follow-up care with pediatric neurology, ophthalmology and otorhinolaryngology specialists.

## Discussion

IFAP syndrome is an another close differential, though it results from a mutation in the MBTPS2 gene and lacks both keratoderma and hearing loss.^2^ Ocular manifestations might appear later in life, but they typically present in 95% of cases, with corneal neovascularization being the most common presentation.^5^ Deficiency of vitamin-D and rickets were found to be associated with KID syndrome.^3^ The most common mutation is D50N missense mutation within the *GJB2* gene.^3^ Heterozygote for the p.Asp50Asn-p.D50N mutation in the GJB2 gene was found to be associated with DWM which is similar in index case.^6^ DWM is a developmental anomaly affecting the midline of the cerebellum, characterized by complete or partial agenesis of the vermis and cystic enlargement of the fourth ventricle and typically manifests with significant neurological dysfunction. However, when it occurs in conjunction with KID syndrome, patients often exhibit no or minimal neurological abnormalities, which aligns with the current case. In previously reported cases, the patients were adults, and they displayed mild symptoms such as ataxia, dizziness, or psychomotor delays.^6^ There is no difference in the clinical manifestation of KID syndrome with or without DWM. MRI or CT scan has been recommended, though DWM is always associated with the p.Asp50Asn-p.D50N mutation or not, is unclear.^6^

To the best of my knowledge, this is the first reported case from India where an infant with KID syndrome, lacking ocular manifestations, sharing a common mutation with the DWM, has been observed. This report emphasizes the significance of investigating additional manifestations of the GJB2 gene mutation in patients with KID syndrome.

## Conflict of interest

None disclosed.
